# Correlation Between Thyroid Function Parameters and Aldosterone–Renin Ratio in Patients With Primary Aldosteronism and Their Association With Target Organ Damage

**DOI:** 10.1155/ije/7275578

**Published:** 2026-07-05

**Authors:** Changjiang Deng, Yixin Xu, Ying Pan, Tingting Wu, Zhihui Jiang, Mingming Lv, Yingying Zheng, Xiang Xie

**Affiliations:** ^1^ Department of Cardiology, The First Affiliated Hospital of Xinjiang Medical University, Urumqi, 830000, Xinjiang, China, xjmu.edu.cn; ^2^ Department of Cardiology, The Southwest Hospital of AMU, Chongqing, 400038, Chongqing, China; ^3^ Department of Pathophysiology, Xinjiang Medical University College of Basic Medicine, Urumqi, 830000, Xinjiang, China

**Keywords:** aldosterone–renin ratio, hypertension, primary aldosteronism, target organ damage, thyroid function

## Abstract

**Background:**

Primary aldosteronism (PA) is one of the most common causes of secondary hypertension. The relationship between thyroid function parameters and the aldosterone–renin ratio (ARR) in PA patients, as well as their combined impact on target organ damage, remains incompletely understood.

**Methods:**

We conducted a retrospective analysis of 317 patients with suspected or confirmed PA who underwent comprehensive endocrine evaluation, including thyroid function testing, plasma aldosterone concentration, plasma renin activity (PRA), ARR, and assessment of target organ damage. Biomarker correlation and regression analyses were performed in the whole cohort, while thyroid function subgroup comparisons were restricted to patients with confirmed PA. Suppressed renin activity was predefined as PRA < 1.0 ng/mL/h, with profound suppression defined as PRA < 0.5 ng/mL/h.

**Results:**

Among 317 patients (54.9% male, 45.1% female), 229 (72.24%) were diagnosed with PA. Hypertension was present in 248 patients (78.23%), of whom 239 (75.39%) were classified as Grade 3 hypertension with very high cardiovascular risk. Thyroid dysfunction was observed in a subset of patients, with elevated TSH levels present among individuals with hypothyroid status. The mean TSH was 3.86 ± 2.41 μIU/mL, and the mean ARR was 48.058 ± 3.161. Significant correlations were found between TSH and ARR (*r* = 0.35, *p* < 0.01). Patients with higher ARR values showed a higher prevalence of target organ damage, including coronary artery atherosclerosis (24.92%), carotid artery atherosclerosis (21.45%), and lacunar cerebral infarction (31.86%). Multiple regression analysis revealed that thyroid function abnormalities and elevated ARR were associated with an increased risk of target organ damage after adjustment for conventional risk factors.

**Conclusion:**

Thyroid function parameters, particularly TSH and FT4, are significantly associated with ARR in patients with suspected or confirmed PA. Thyroid dysfunction and elevated ARR are associated with target organ damage, and their combined assessment may improve endocrine and cardiovascular risk stratification in PA‐related hypertension.

## 1. Introduction

Primary aldosteronism (PA) represents a group of disorders characterized by autonomous aldosterone overproduction from the adrenal glands, independent of the renin–angiotensin system [1]. Once considered a rare cause of hypertension, PA is now recognized as the most common form of secondary hypertension, with prevalence estimates ranging from 5% to 20% among hypertensive patients [2]. PA is typically diagnosed using screening tests that measure plasma aldosterone concentration (PAC), plasma renin activity (PRA), and their ratio (aldosterone‐to‐renin ratio (ARR)), followed by confirmatory tests and adrenal imaging [3].

The clinical significance of PA extends beyond blood pressure elevation, as patients with PA demonstrate disproportionate target organ damage compared to essential hypertensive patients with equivalent blood pressure levels [4]. This excess damage is attributed to aldosterone’s direct proinflammatory and profibrotic effects on various tissues, including the cardiovascular system, kidneys, and brain, independent of its effects on blood pressure [5]. Cardiovascular complications of PA include left ventricular hypertrophy, atrial fibrillation, coronary artery disease, and heart failure, while renal manifestations include albuminuria and progressive renal dysfunction [6].

The renin–angiotensin–aldosterone system (RAAS) interacts with multiple endocrine axes, including the hypothalamic–pituitary–thyroid axis. Thyroid hormones play crucial roles in cardiovascular homeostasis, influencing cardiac contractility, heart rate, vascular resistance, and blood pressure [7]. Thyroid dysfunction, even subclinical, has been associated with increased cardiovascular morbidity and mortality [8]. The interplay between thyroid function and RAAS is complex and bidirectional. Experimental studies have demonstrated that thyroid hormones can modulate renin gene expression and RAAS activity, while aldosterone may influence thyroid hormone metabolism and action [9].

Despite these established physiological connections, the clinical relationship between thyroid function parameters and the ARR in patients with PA remains incompletely characterized. Limited studies have explored how the thyroid function might influence the diagnosis and phenotype of PA, with some suggesting that thyroid dysfunction may alter ARR values and potentially lead to misdiagnosis [10]. However, a comprehensive analysis of multiple thyroid function parameters and their relationship with ARR in a large cohort of PA patients is lacking.

Furthermore, the potential contribution of thyroid dysfunction to target organ damage in PA patients represents an important knowledge gap. While both PA and thyroid disorders independently increase cardiovascular risk, their combined impact may be synergistic rather than merely additive. Elucidating this relationship could have important implications for risk stratification and management of PA patients [11].

Another consideration is the potential ethnic variation in the presentation and complications of PA. Most large‐scale studies on PA have been conducted in Western populations, with fewer comprehensive analyses in Asian populations, particularly those with diverse ethnic compositions [12]. Given potential genetic and environmental differences that may influence both PA and thyroid function, population‐specific data are essential for optimizing clinical care.

The interaction between PA and thyroid dysfunction may also have therapeutic implications. Treatment approaches for PA include mineralocorticoid receptor antagonists (MRAs) and adrenalectomy for unilateral disease. Whether thyroid function parameters influence treatment outcomes or should guide therapeutic decisions remains uncertain. Similarly, whether correcting thyroid dysfunction might ameliorate some manifestations of PA or reduce target organ damage has not been systematically evaluated.

The diagnostic criteria and management approaches for PA have evolved significantly over recent decades, but substantial clinical challenges persist. These include accurate disease classification, prediction of the risk of target organ damage, and personalized treatment selection. Incorporating additional biomarkers, such as thyroid function parameters, into clinical assessment might improve patient stratification and management [3].

Recent technological advances in laboratory medicine have enabled more precise measurement of hormones and their metabolites, facilitating a more nuanced understanding of endocrine pathophysiology. Application of these technologies to explore the relationship between thyroid function and the RAAS may reveal previously unrecognized interactions and disease mechanisms [5].

The present study aims to address these knowledge gaps by analyzing the relationship between thyroid function parameters (TSH, FT3, FT4, TT3, TT4, TPO‐Ab, TG‐Ab) and ARR in a large cohort of patients with PA and by examining how these factors, individually and jointly, contribute to target organ damage.

## 2. Methods

### 2.1. Study Design and Population

#### 2.1.1. Study Design

We conducted a retrospective analysis of patients diagnosed with or suspected of PA who were admitted to our medical center between January 2020 and December 2024. The study was approved by the Institutional Review Board, and the requirement for individual informed consent was waived due to the retrospective nature of the analysis. All procedures were performed in accordance with the ethical standards of the institutional research committee and the 1964 Declaration of Helsinki and its later amendments.

#### 2.1.2. Patient Selection

Inclusion criteria were (1) age ≥ 18 years; (2) suspected or confirmed diagnosis of PA based on clinical presentation, laboratory tests, or imaging findings; and (3) availability of complete clinical, laboratory, and imaging data. The overall cohort consisted of 317 patients referred to for PA evaluation. Among them, 229 met confirmatory diagnostic criteria for PA, whereas 88 were retained as a nonconfirmed suspected PA/low‐renin hypertension comparator group for describing the overall biomarker distribution. Analyses of thyroid status subclasses were performed within the confirmed PA cohort to avoid conflating confirmed PA with nonconfirmed low‐renin hypertension phenotypes.

Patients were regarded as “suspected PA” if they had an ARR ≥ 20 ng/dL per ng mL^−1^ h^−1^ and PAC ≥ 15 ng/dL on at least one outpatient measurement. Suppression of renin activity was predefined as PRA < 1.0 ng/mL/h, and profound renin suppression was defined as PRA < 0.5 ng/mL/h. Complete medical records referred to the index hospitalization and baseline laboratory/imaging data; longitudinal follow‐up was not mandatory for study entry.

### 2.2. Clinical and Laboratory Assessments

#### 2.2.1. Demographic and Clinical Data Collection

Demographic information (age, sex, ethnicity, occupation) and clinical data, including medical history, physical examination findings, and medication use, were extracted from electronic medical records. Blood pressure measurements were obtained using standardized procedures, with hypertension classified according to the 2018 ESC/ESH Guidelines for the management of arterial hypertension. Medication records primarily reflected treatments administered during hospitalization for diagnostic evaluation and electrolyte correction rather than long‐term outpatient pharmacotherapy.

#### 2.2.2. Biochemical Analysis

All patients underwent a comprehensive biochemical evaluation after appropriate preparation. Blood samples were collected in the morning after overnight fasting. For hormonal assessments, patients discontinued interfering medications according to established guidelines: MRAs for at least 4 weeks; diuretics, beta‐blockers, angiotensin‐converting enzyme inhibitors, and angiotensin receptor blockers for at least 2 weeks, when possible.

Serum electrolytes, including potassium (K), were measured using ion‐selective electrode methods. Renal function was assessed by measuring serum creatinine using the Jaffe method. Lipid profile parameters, including total cholesterol (TC), triglycerides (TGs), high‐density lipoprotein cholesterol (HDL‐C), and low‐density lipoprotein cholesterol (LDL‐C), were determined using enzymatic colorimetric methods. Glycated hemoglobin (HbA1c) was measured using high‐performance liquid chromatography.

#### 2.2.3. Hormonal Measurements

Plasma aldosterone concentration (ALD) was measured using radioimmunoassay with commercially available kits. PRA was determined by radioimmunoassay, measuring angiotensin I generation. The ARR was calculated by dividing PAC by PRA.

Thyroid function was assessed by measuring thyroid‐stimulating hormone (TSH), free triiodothyronine (FT3), free thyroxine (FT4), total triiodothyronine (TT3), total thyroxine (TT4), thyroid peroxidase antibodies (TPO‐Ab), and thyroglobulin antibodies (TG‐Ab) using chemiluminescent immunoassay methods. Thyroid functional status was classified according to established clinical reference ranges into euthyroid, subclinical hypothyroidism, overt hypothyroidism, subclinical hyperthyroidism, and overt hyperthyroidism.

N‐terminal pro‐B‐type natriuretic peptide (NT‐proBNP) was measured as a marker of cardiac loading status. Myoglobin and creatine kinase (CK) were recorded as routine biochemical variables but were not used to define cardiovascular target organ damage because the primary cardiovascular damage definition was based on structural, electrocardiographic, and clinically documented coronary outcomes. High‐sensitivity cardiac troponin was not consistently available in the retrospective database and was therefore not incorporated into the main analyses.

#### 2.2.4. Diagnostic Criteria for PA

The diagnosis of PA was based on the Endocrine Society Clinical Practice Guidelines. Patients with elevated ARR (> 20 ng/dL per ng/mL/h) and PAC (> 15 ng/dL) underwent confirmatory testing using the saline infusion test or captopril challenge test when clinically appropriate. The diagnosis was confirmed when plasma aldosterone failed to suppress appropriately during confirmatory testing, indicating inappropriate aldosterone production.

### 2.3. Imaging Studies

#### 2.3.1. Cardiac Imaging

Left ventricular end‐diastolic diameter (LVEDD) and interventricular septal (IVS) thickness were measured by transthoracic echocardiography. Left ventricular ejection fraction (LVEF) was calculated using the modified Simpson’s method. Left ventricular hypertrophy was defined according to established echocardiographic criteria.

#### 2.3.2. Adrenal Imaging

All patients underwent adrenal imaging using either computed tomography (CT) or magnetic resonance imaging (MRI) to identify potential adrenal adenomas, hyperplasia, or other abnormalities. CT or MRI protocols included noncontrast and contrast‐enhanced sequences with thin‐slice reconstruction of the adrenal glands.

#### 2.3.3. Cerebrovascular Imaging

Brain MRI was performed to evaluate cerebrovascular damage, including lacunar infarctions and white matter lesions. Carotid ultrasound was performed to assess carotid artery atherosclerosis, with measurements of intima‐media thickness and plaque characterization.

### 2.4. Definition of Target Organ Damage

#### 2.4.1. Cardiovascular Damage

Cardiovascular damage was defined using prespecified structural, electrocardiographic, and clinically documented coronary criteria, rather than nonspecific muscle enzyme markers.

#### 2.4.2. Cerebrovascular Damage

Cerebrovascular damage was defined as the presence of one or more of the following: lacunar cerebral infarction (identified on brain MRI), carotid artery atherosclerosis (> 50% stenosis or the presence of atherosclerotic plaques on carotid ultrasound), and history of stroke or transient ischemic attack.

#### 2.4.3. Renal Damage

Renal damage was defined as the presence of one or more of the following: albuminuria (urinary albumin‐to‐creatinine ratio > 30 mg/g), reduced estimated glomerular filtration rate (< 60 mL/min/1.73 m^2^), or renal artery stenosis confirmed by imaging.

### 2.5. Statistical Analysis

#### 2.5.1. Descriptive Statistics

Continuous variables with normal distribution were presented as mean ± standard deviation, while non‐normally distributed variables were presented as median and interquartile range. Categorical variables were expressed as absolute numbers and percentages. The Kolmogorov–Smirnov test was used to assess the normality of continuous variables.

#### 2.5.2. Comparative Analysis

Patients were categorized into subgroups based on thyroid function status (euthyroid, subclinical hypothyroidism, subclinical hyperthyroidism, overt hypothyroidism, and overt hyperthyroidism) and ARR tertiles (low < 34, middle 34–62, high > 62). The distribution of baseline characteristics was first summarized for the whole cohort, whereas thyroid function subgroup comparisons were restricted to confirmed PA patients. Differences between groups were assessed using one‐way analysis of variance (ANOVA) or Kruskal–Wallis test for continuous variables, and chi‐squared test or Fisher’s exact test for categorical variables, as appropriate.

#### 2.5.3. Correlation and Regression Analysis

Pearson’s or Spearman’s correlation coefficients were calculated to evaluate relationships between thyroid function parameters and ARR. Correlation and regression analyses of ARR were performed in the whole cohort to characterize the full spectrum of referred patients, and subgroup analyses among confirmed PA patients were used to evaluate clinical phenotype differences according to thyroid status. Multiple linear regression analyses were performed to identify independent predictors of ARR, adjusting for potential confounders, including age, sex, body mass index, blood pressure, and medication use.

Logistic regression models were constructed to assess the associations between thyroid function parameters, ARR, and target organ damage, adjusting for traditional cardiovascular risk factors.

In regression models evaluating renal target organ damage, diabetes mellitus status was additionally included as a covariate to reduce potential confounding effects on albuminuria. Because overt hypo‐ and hyperthyroid subgroups were small, analyses evaluating the combined association of thyroid dysfunction and ARR with target organ damage were interpreted as exploratory and hypothesis‐generating rather than definitive causal estimates.

Odds ratios (ORs) and 95% confidence intervals (CIs) were calculated.

#### 2.5.4. Statistical Software

All statistical analyses were performed using SPSS Statistics version 25.0 (IBM Corp., Armonk, NY, USA) and R version 4.0.3 (R Foundation for Statistical Computing, Vienna, Austria). Two‐tailed *p* values < 0.05 were considered statistically significant.

## 3. Results

### 3.1. Baseline Characteristics of the Study Population

A total of 317 patients meeting the study criteria were included in the analysis. The study population comprised 174 men (54.9%) and 143 women (45.1%), with a diverse ethnic background including Han (227, 71.61%), Uyghur (54, 17.03%), Hui (17, 5.36%), Kazakh (12, 3.79%), and other ethnic background minorities. The occupational distribution included office workers (86, 27.13%), retirees (62, 19.56%), professional technicians (45, 14.20%), self‐employed individuals (30, 9.46%), and government employees (28, 8.83%).

The baseline demographic and clinical characteristics of the 317 patients included in this study are presented in Table 1, while biochemical, hormonal, and echocardiographic characteristics are presented separately in Table 2 to improve readability. The mean age of the cohort was 54.3 ± 12.5 years, with a male predominance. The ethnic distribution reflected the regional demographics, with Han Chinese constituting the majority (71.61%), followed by Uyghur (17.03%), Hui (5.36%), and Kazakh (3.79%).

**TABLE 1:** Baseline demographic and clinical characteristics of the study population (*N* = 317).

Characteristic	Value
Age (years), mean ± SD	54.3 ± 12.5
Male, *n* (%)	174 (54.9)
Female, *n* (%)	143 (45.1)
Han ethnicity, *n* (%)	227 (71.61)
Uyghur ethnicity, *n* (%)	54 (17.03)
Hui ethnicity, *n* (%)	17 (5.36)
Kazakh ethnicity, *n* (%)	12 (3.79)
Other ethnic background, *n* (%)	7 (2.21)
Office workers, *n* (%)	86 (27.13)
Retirees, *n* (%)	62 (19.56)
Professional technicians, *n* (%)	45 (14.20)
Self‐employed individuals, *n* (%)	30 (9.46)
Government employees, *n* (%)	28 (8.83)
Other occupations, *n* (%)	66 (20.82)
Hypertension, *n* (%)	248 (78.23)
Grade 3 hypertension, *n* (%)	239 (75.39)
Confirmed primary aldosteronism, *n* (%)	229 (72.24)
Nonconfirmed suspected PA/low‐renin hypertension phenotype, *n* (%)	88 (27.76)
Fatty liver, *n* (%)	158 (49.84)
Chronic cholecystitis, *n* (%)	149 (47.00)
Hypokalemia, *n* (%)	120 (37.85)
Nontoxic thyroid nodule, *n* (%)	104 (32.81)
Thyroid nodules, *n* (%)	95 (29.97)
Lacunar cerebral infarction, *n* (%)	101 (31.86)
Coronary atherosclerotic heart disease, *n* (%)	79 (24.92)
Carotid artery atherosclerosis, *n* (%)	68 (21.45)
Type 2 diabetes mellitus, *n* (%)	62 (19.56)

**TABLE 2:** Baseline biochemical, hormonal, and echocardiographic characteristics of the study population (*N* = 317).

Characteristic	Value
Creatinine (μmol/L)	79.6 ± 18.4
Total cholesterol (mmol/L)	1.67 ± 0.92
Triglycerides (mmol/L)	1.21 ± 0.36
HDL‐C (mmol/L)	1.21 ± 0.36
LDL‐C (mmol/L)	3.12 ± 0.88
Potassium (mmol/L)	3.42 ± 0.54
TSH (μIU/mL)	3.86 ± 2.41
FT4 (pmol/L)	14.8 ± 3.6
FT3 (pmol/L)	4.72 ± 1.18
TT3 (nmol/L)	1.73 ± 0.41
TT4 (nmol/L)	97.4 ± 21.6
TG‐Ab (IU/mL)	34.5 ± 72.1
TPO‐Ab (IU/mL)	12.3 ± 18.7
HbA1c (%)	6.4 ± 1.2
Aldosterone (ng/dL)	23.5 ± 14.2
PRA (ng/mL/h)	0.56 ± 0.41
ARR	48.058 ± 3.161
NT‐proBNP (pg/mL)	124.5 ± 98.3
LVEDD (mm)	50.6 ± 5.4
IVS (mm)	11.8 ± 2.3

*Note:* Continuous variables are presented as mean ± standard deviation (SD), and categorical variables as counts (percentages). FT3, free triiodothyronine; FT4, free thyroxine; TT3, total triiodothyronine; TG‐Ab, thyroglobulin antibody; TPO‐Ab, thyroid peroxidase antibody; TT4, total thyroxine.

Abbreviations: ARR, aldosterone–renin ratio; HbA1c, glycated hemoglobin; IVS, interventricular septal thickness; LVEDD, left ventricular end‐diastolic diameter; NT‐proBNP, N‐terminal pro‐B‐type natriuretic peptide; PRA, plasma renin activity; TSH, thyroid‐stimulating hormone.

PA was confirmed in 229 patients (72.24%), while 88 patients remained in the nonconfirmed suspected PA/low‐renin hypertension comparator group. Hypertension was present in 248 patients (78.23%). A substantial proportion of patients had grade 3 hypertension with very high cardiovascular risk (239, 75.39%). Common comorbidities included fatty liver (158, 49.84%), chronic cholecystitis (149, 47.00%), hypokalemia (120, 37.85%), thyroid nodules (95, 29.97%), and type 2 diabetes mellitus (62, 19.56%). Evidence of target organ damage was also common, with 101 patients (31.86%) showing lacunar cerebral infarction, 79 (24.92%) diagnosed with coronary atherosclerotic heart disease, and 68 (21.45%) demonstrating carotid artery atherosclerosis.

Laboratory evaluation demonstrated biochemical features consistent with PA, including a relatively high ARR and frequent hypokalemia (Table 2). Thyroid dysfunction was present in a subset of patients. Although the overall cohort included individuals with elevated TSH levels, the majority of patients were euthyroid, and the distribution of TSH was influenced by a limited number of patients with overt hypothyroidism.

### 3.2. Correlation Between Thyroid Function Parameters and ARR

**Figure 1:**
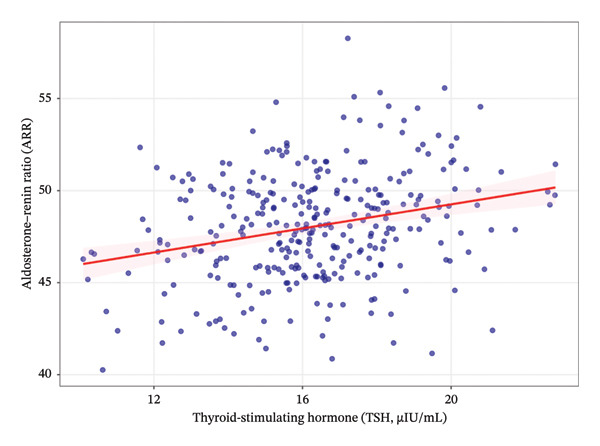
Correlation between thyroid‐stimulating hormone and aldosterone–renin ratio scatter plot demonstrating a significant positive correlation between thyroid‐stimulating hormone (TSH) and the aldosterone–renin ratio (ARR) in the study population (*r* = 0.35, *p* < 0.01).

To investigate the relationship between thyroid function and the RAAS, we analyzed correlations between various thyroid parameters and ARR. Figure 1 illustrates the significant positive correlation between TSH and ARR (*r* = 0.35, *p* < 0.01), indicating that higher TSH levels were associated with higher ARR values. Because TSH distribution was influenced by a limited number of patients with overt hypothyroidism, these findings were interpreted in conjunction with thyroid function subgroup analyses.

**Figure 2:**
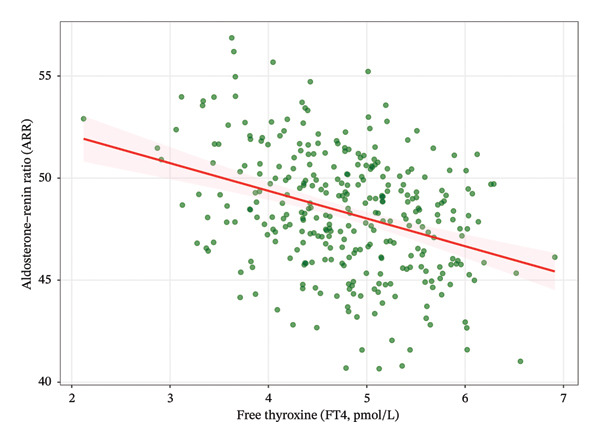
Correlation between free thyroxine and aldosterone–renin ratio scatter plot illustrating a significant negative correlation between free thyroxine (FT4) levels and the aldosterone–renin ratio (ARR), indicating that lower FT4 levels are associated with higher ARR values (*r* = −0.28, *p* < 0.01).

Conversely, FT4 showed a significant negative correlation with ARR (*r* = −0.28, *p* < 0.01), as depicted in Figure 2. This inverse relationship suggests that lower free T4 levels are associated with higher ARR values.

Further analysis of other thyroid parameters revealed a significant negative correlation between FT3 and ARR (*r* = −0.22, *p* < 0.01), while TT3 and TT4 showed weaker correlations with ARR. Thyroid autoantibodies (TPO‐Ab and TG‐Ab) did not demonstrate significant correlations with ARR in our cohort.

Table 3 presents the results of this analysis, confirming that TSH (*β* = 0.31, *p* < 0.01) and FT4 (*β* = −0.24, *p* < 0.01) remained independently associated with ARR after adjustment for confounders.

**TABLE 3:** Multiple linear regression analysis for predictors of aldosterone–renin ratio.

Variable	Standardized β	95% CI	*p* value
TSH	0.31	0.18 to 0.44	< 0.01
FT4	−0.24	−0.37 to −0.11	< 0.01
FT3	−0.18	−0.31 to −0.05	0.02
Age	0.15	0.02 to 0.28	0.03
Female sex	0.12	−0.01 to 0.25	0.06
BMI	0.10	−0.03 to 0.23	0.09
Systolic BP	0.24	0.11 to 0.37	< 0.01
Diastolic BP	0.17	0.04 to 0.30	0.02
Potassium	−0.29	−0.42 to −0.16	< 0.01

*Note:* Standardized β coefficients represent the strength and direction of association between each variable and the aldosterone–renin ratio (ARR) after adjustment for other variables in the regression model. FT3, free triiodothyronine; FT4, free thyroxine.

Abbreviations: BMI, body mass index; BP, blood pressure; CI, confidence interval; TSH, thyroid‐stimulating hormone.

### 3.3. Thyroid Function Status and Clinical Characteristics in PA

Table 4 compares the clinical and biochemical characteristics across these groups. Because the overt hypothyroidism, subclinical hyperthyroidism, and overt hyperthyroidism subgroups were small, between‐group estimates were interpreted cautiously and used primarily to describe trends across thyroid function strata.

**TABLE 4:** Clinical and biochemical characteristics of PA patients by thyroid function.

Characteristic	Euthyroid (*n* = 152)	Subclinical hypothyroidism (*n* = 53)	Overt hypothyroidism (*n* = 10)	Subclinical hyperthyroidism (*n* = 9)	Overt hyperthyroidism (*n* = 5)	*p* value
Age (years)	53.8 ± 11.7	56.2 ± 12.3	59.1 ± 10.5	52.4 ± 13.1	49.8 ± 15.2	0.08
Female, *n* (%)	67 (44.1)	28 (52.8)	6 (60.0)	4 (44.4)	3 (60.0)	0.14
SBP (mmHg)	162.3 ± 22.7	168.5 ± 25.6	174.9 ± 21.3	161.8 ± 23.4	166.2 ± 27.8	0.03
DBP (mmHg)	98.7 ± 14.2	102.3 ± 15.6	105.8 ± 13.7	97.5 ± 16.1	99.2 ± 18.4	0.04
Potassium (mmol/L)	3.24 ± 0.53	3.02 ± 0.61	2.85 ± 0.48	3.35 ± 0.58	3.28 ± 0.62	< 0.01
Creatinine (μmol/L)	78.6 ± 19.8	81.5 ± 22.3	86.2 ± 24.5	77.8 ± 21.2	79.3 ± 23.7	0.21
Aldosterone (ng/dL)	25.3 ± 11.8	28.7 ± 13.2	32.4 ± 14.5	24.8 ± 12.6	26.5 ± 13.1	0.03
PRA (ng/mL/h)	0.59 ± 0.42	0.46 ± 0.38	0.35 ± 0.29	0.62 ± 0.45	0.58 ± 0.40	< 0.01
ARR	45.2 ± 26.7	58.6 ± 32.8	69.3 ± 35.7	42.3 ± 24.5	48.7 ± 28.3	< 0.01
LVEDD (mm)	48.2 ± 5.6	49.7 ± 6.2	51.3 ± 5.8	47.8 ± 5.9	48.5 ± 6.3	0.07
IVS (mm)	11.2 ± 2.1	12.1 ± 2.3	13.5 ± 2.4	11.0 ± 2.2	11.4 ± 2.1	< 0.01

*Note:* Continuous variables are presented as mean ± SD, and categorical variables as numbers (percentages). Group comparisons were performed using analysis of variance (ANOVA) for continuous variables and the *χ* ^2^ test for categorical variables.

Abbreviations: ARR, aldosterone–renin ratio; DBP, diastolic blood pressure; IVS, interventricular septal thickness; LVEDD, left ventricular end‐diastolic diameter; PRA, plasma renin activity; SBP, systolic blood pressure.

Patients with hypothyroidism, including both subclinical and overt hypothyroidism, demonstrated significantly higher blood pressure, lower potassium levels, higher aldosterone, lower PRA, and consequently higher ARR compared with euthyroid patients. In addition, IVS thickness was significantly greater in hypothyroid patients, suggesting more pronounced cardiac remodeling.

**Figure 3:**
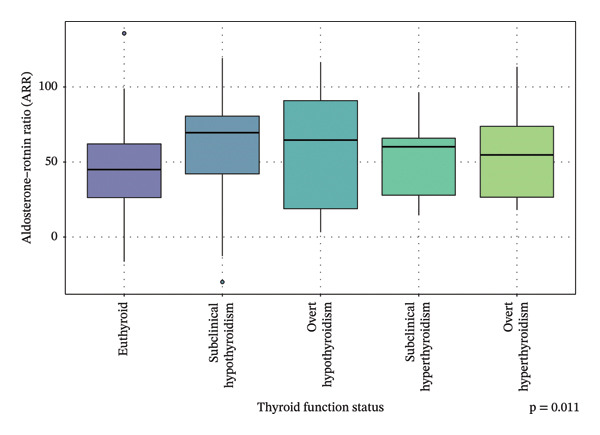
Comparison of aldosterone–renin ratio across thyroid function groups. Box‐and‐whisker plot comparing ARR values among euthyroid, subclinical hypothyroid, overt hypothyroid, subclinical hyperthyroid, and overt hyperthyroid groups. Patients with hypothyroidism demonstrated significantly higher ARR values (*p* < 0.01).

These findings are visualized in Figure 3, which presents a box plot comparison of ARR across different thyroid function groups and demonstrates elevated ARR in hypothyroid patients.

### 3.4. Association of Thyroid Function Parameters and ARR With Target Organ Damage

**Figure 4:**
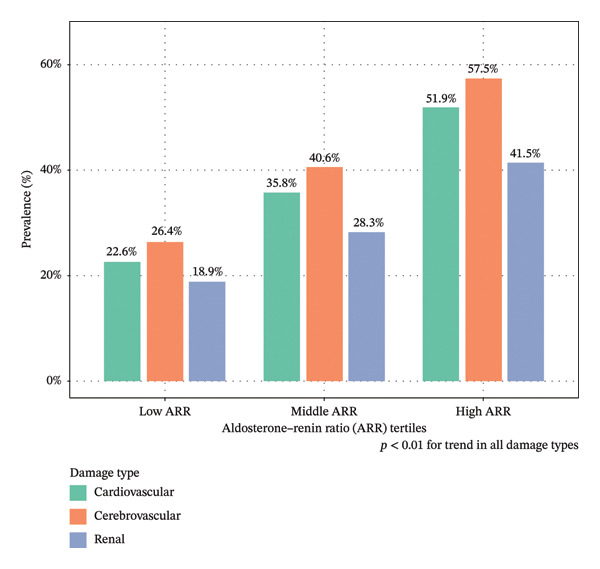
Prevalence of target organ damage across ARR tertile bar graph showing the prevalence of cardiovascular, cerebrovascular, and renal target organ damage across increasing tertiles of the aldosterone–renin ratio (ARR). A progressive increase in the prevalence of organ damage was observed with higher ARR levels (*p* < 0.01 for trend).

To evaluate the clinical significance of the observed correlations between thyroid function and ARR, we analyzed their association with various forms of target organ damage. Figure 4 illustrates the prevalence of cardiovascular, cerebrovascular, and renal damage across tertiles of ARR.

The prevalence of all forms of target organ damage increased progressively with higher ARR tertiles. Cardiovascular damage, including coronary artery disease and left ventricular hypertrophy, was present in 22.6%, 35.8%, and 51.9% of patients in the low, middle, and high ARR tertiles, respectively (*p* < 0.01 for trend). Similarly, cerebrovascular damage, including lacunar infarction and carotid atherosclerosis, showed increasing prevalence across ARR tertiles: 26.4%, 40.6%, and 57.5%, respectively (*p* < 0.01 for trend). Renal damage demonstrated a similar pattern, with prevalence rates of 18.9%, 28.3%, and 41.5% across ARR tertiles (*p* < 0.01 for trend).

**Figure 5:**
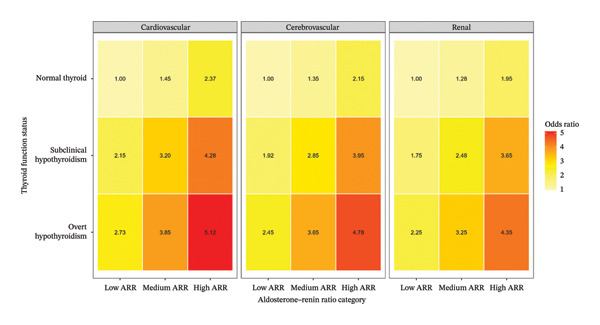
Combined impact of thyroid dysfunction and ARR on target organ damage matrix plot presenting adjusted odds ratios for cardiovascular, cerebrovascular, and renal target organ damage according to thyroid function status and ARR categories. Patients with both hypothyroidism and elevated ARR showed the highest risk of organ damage.

We further examined the combined association of thyroid dysfunction and elevated ARR with target organ damage. Figure 5 presents the odds ratios for target organ damage according to the thyroid function status and ARR categories in a matrix format. Patients with both hypothyroidism and high ARR demonstrated higher odds of target organ damage compared with those with either condition alone. For cardiovascular damage, the adjusted odds ratio was 4.28 (95% CI, 2.86–6.42) for patients with both conditions, compared with 2.15 (95% CI, 1.42–3.26) for hypothyroidism alone and 2.37 (95% CI, 1.58–3.56) for high ARR alone. Similar patterns were observed for cerebrovascular and renal damage; however, these combined subgroup results were considered exploratory because of the small number of patients in several thyroid dysfunction categories.

Table 5 presents the results of this analysis.

**TABLE 5:** Multivariate logistic regression analysis for predictors of target organ damage.

Predictor	Cardiovascular damage OR (95% CI)	*p* value	Cerebrovascular damage OR (95% CI)	*p* value	Renal damage OR (95% CI)	*p* value
Age (per 10 years)	1.42 (1.21–1.67)	< 0.01	1.58 (1.35–1.85)	< 0.01	1.31 (1.12–1.54)	< 0.01
Male sex	1.28 (0.86–1.91)	0.22	1.15 (0.78–1.70)	0.48	1.45 (0.97–2.16)	0.07
Smoking	1.53 (1.02–2.31)	0.04	1.36 (0.91–2.03)	0.13	1.22 (0.81–1.83)	0.35
Diabetes	1.77 (1.14–2.74)	0.01	1.62 (1.06–2.49)	0.03	2.15 (1.41–3.28)	< 0.01
LDL‐C (per mmol/L)	1.48 (1.16–1.89)	< 0.01	1.37 (1.08–1.74)	0.01	1.19 (0.94–1.51)	0.15
TSH (per 5 μIU/mL)	1.29 (1.08–1.55)	< 0.01	1.23 (1.03–1.47)	0.02	1.18 (0.98–1.42)	0.08
FT4 (per pmol/L)	0.82 (0.71–0.95)	< 0.01	0.87 (0.75–1.00)	0.05	0.90 (0.78–1.04)	0.16
ARR (per 10 units)	1.26 (1.12–1.41)	< 0.01	1.22 (1.09–1.37)	< 0.01	1.19 (1.06–1.34)	< 0.01
SBP (per 10 mmHg)	1.18 (1.06–1.31)	< 0.01	1.16 (1.04–1.29)	< 0.01	1.21 (1.09–1.35)	< 0.01
Potassium (per mmol/L)	0.68 (0.53–0.87)	< 0.01	0.75 (0.59–0.96)	0.02	0.62 (0.48–0.80)	< 0.01

*Note:* Odds ratios were obtained from multivariate logistic regression models adjusted for potential confounders. Values greater than one indicate increased risk, whereas values less than one indicate a protective association with the outcome. FT4, free thyroxine.

Abbreviations: ARR, aldosterone–renin ratio; CI, confidence interval; LDL‐C, low‐density lipoprotein cholesterol; OR, odds ratio; SBP, systolic blood pressure; TSH, thyroid‐stimulating hormone.

Thyroid function parameters, particularly TSH and FT4, and ARR were associated with target organ damage after adjustment for traditional risk factors. Higher TSH, lower FT4, and higher ARR were independently associated with increased odds of cardiovascular and cerebrovascular damage, whereas for renal damage, the association remained statistically significant for ARR but not for thyroid parameters.

**Figure 6:**
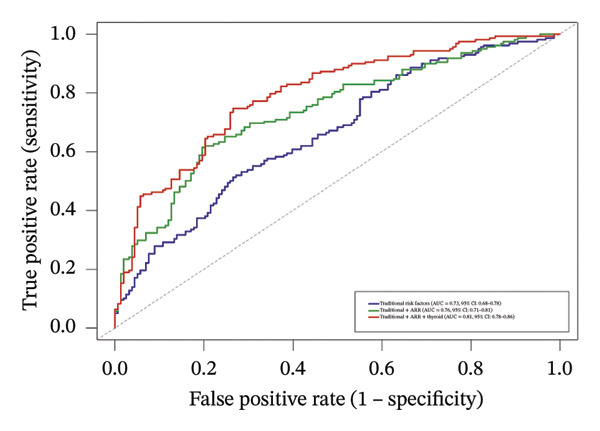
Predictive performance of combined models for target organ damage. Receiver operating characteristic (ROC) curves comparing predictive models for target organ damage using traditional risk factors alone, traditional risk factors plus ARR, and a combined model including ARR and thyroid parameters. The combined model demonstrated improved predictive performance.

Figure 6 illustrates the receiver operating characteristic (ROC) curves for predicting target organ damage across different combinations of predictors. The area under the curve (AUC) for prediction of cardiovascular damage was 0.73 (95% CI 0.68–0.78) for traditional risk factors alone, 0.76 (95% CI 0.71–0.81) when adding ARR, and 0.81 (95% CI 0.76–0.86) when incorporating both ARR and thyroid parameters, indicating improved predictive performance with the combined model.

### 3.5. Medication Use and Treatment Response

During hospitalization, several supportive treatments were administered as part of routine diagnostic preparation and electrolyte correction. The most common interventions included potassium chloride supplementation, intravenous saline infusion, and spironolactone therapy. Recorded oral glucose administration primarily reflected in‐hospital metabolic management or testing‐related use rather than routine treatment for diabetes mellitus.

We further evaluated whether medication exposure influenced the observed relationship between thyroid function parameters and ARR. Patients receiving spironolactone had lower ARR values than those not receiving MRAs; however, the correlation between thyroid function parameters and ARR remained significant in both groups, suggesting that the observed relationship was not solely attributable to medication effects.

in which ARR normalized in 78% of patients, and the left ventricular mass index decreased by 15.6% (*p* < 0.001). Because follow‐up data were incomplete and treatment allocation was not randomized, these longitudinal observations were interpreted descriptively.

## 4. Discussion

In this retrospective analysis of 317 patients referred for evaluation of suspected or confirmed PA, we demonstrated significant correlations between thyroid function parameters and the ARR, as well as associations with target organ damage. The biomarker correlation analysis was performed in the whole referred cohort, whereas thyroid function subgroup comparisons were focused on the 229 patients with confirmed PA. This distinction clarifies the study population and avoids conflating confirmed PA with nonconfirmed suspected PA or low‐renin hypertension phenotypes.

This suggests a clinically relevant association between the hypothalamic–pituitary–thyroid axis and the RAAS. Importantly, thyroid dysfunction, especially hypothyroidism, and elevated ARR were associated with an increased risk of target organ damage, with an apparent additive pattern when both conditions coexisted [10–12]. Because several thyroid dysfunction subclasses contained relatively few patients, the combined‐risk analysis should be regarded as hypothesis‐generating rather than definitive evidence of a causal interaction.

The observed correlation between the thyroid function and ARR has important clinical implications. Elevated ARR is the primary screening test for PA, and our findings suggest that thyroid dysfunction, particularly hypothyroidism, may influence ARR values and should be considered when interpreting ARR in clinically complex patients. This is consistent with previous studies by Sang et al. [13] and Maiturouzi et al. [14], who reported altered aldosterone–renin relationships in patients with thyroid disorders. However, our study extends these observations by demonstrating correlations across the spectrum of thyroid function parameters and by quantifying their association with ARR in a large cohort that included a confirmed PA subgroup.

The mechanisms underlying the observed correlations warrant consideration. Thyroid hormones influence cardiovascular hemodynamics through multiple pathways, including direct effects on cardiac contractility and systemic vascular resistance [15]. These hemodynamic changes may indirectly affect renin secretion by altering renal perfusion pressure. Additionally, thyroid hormones may directly modulate renin gene expression, as demonstrated in experimental studies by Ichihara et al. [16], who identified thyroid hormone response elements in the renin gene promoter.

Conversely, RAAS components may influence thyroid function. Aldosterone has been shown to modulate iodide transport in thyroid cells [17], potentially affecting thyroid hormone synthesis. Furthermore, angiotensin II receptors are expressed in thyroid tissue, and angiotensin II may influence thyroid blood flow and function [18]. These bidirectional interactions create a complex interplay between thyroid function and the RAAS, consistent with our observed correlations.

The association between thyroid dysfunction, elevated ARR, and target organ damage represents a key finding of our study. Both conditions were associated with cardiovascular, cerebrovascular, and renal damage, with an apparent additive pattern when they coexisted. These findings suggest that thyroid dysfunction may represent an additional modifier of clinical risk in PA patients, although prospective interventional studies are needed to confirm causality and quantify the independent contribution of each endocrine abnormality.

Several data characteristics identified in our cohort require clarification. First, the presence of elevated TSH values reflects the inclusion of patients with hypothyroid conditions rather than a systematic feature of PA itself. Second, renal damage assessment using albuminuria may be influenced by diabetes mellitus; therefore, diabetes status was included as a covariate in the multivariable models. Third, electrolyte abnormalities, especially hypokalemia, are common in PA and help explain the lower potassium values observed in our population. Fourth, high‐sensitivity cardiac troponin was not consistently available in the retrospective database; therefore, cardiovascular damage was defined using echocardiographic, electrocardiographic, and clinically documented coronary criteria rather than myoglobin or CK.

Hypothyroid patients demonstrated increased IVS thickness and a higher prevalence of left ventricular hypertrophy compared with euthyroid individuals, even after adjusting for blood pressure differences. These findings are compatible with the broader literature linking thyroid dysfunction and hypertension to adverse cardiac remodeling. In the context of PA, aldosterone‐mediated fibrosis may interact with thyroid hormone–dependent cardiac remodeling, although the small number of patients with overt thyroid dysfunction limits firm inference about effect modification.

Cerebrovascular damage, including lacunar infarction and carotid atherosclerosis, was also independently associated with both thyroid dysfunction and elevated ARR. The cerebrovascular effects of aldosterone excess are well documented [19], but the additional impact of thyroid dysfunction has received less attention. Our findings suggest that thyroid status should be considered when assessing cerebrovascular risk in PA patients, particularly given the high prevalence of lacunar infarction (31.86%) in our cohort.

Renal damage showed stronger associations with ARR than with thyroid parameters in our analysis. This may reflect the direct effects of aldosterone on renal tissue, including the promotion of inflammation, fibrosis, and podocyte injury [20]. However, the trend toward increased renal damage with thyroid dysfunction suggests that thyroid hormones may modulate aldosterone’s renal effects or independently influence renal structure and function.

The ethnic diversity of our cohort enabled the exploration of potential ethnic variations in the relationship among thyroid function, ARR, and target organ damage. Although we did not observe significant differences in the strength of correlations across ethnic groups, the high prevalence of PA in our population (72.24%) is notable and consistent with previous reports of increased PA prevalence in Asian populations [21]. This underscores the importance of PA screening in hypertensive patients, particularly in diverse ethnic contexts.

Our study has several strengths, including a relatively large referred cohort, a comprehensive assessment of multiple thyroid parameters, a detailed evaluation of target organ damage, and a diverse ethnic composition. The separation of the overall referred cohort from the confirmed PA subgroup also strengthens the interpretability of the biomarker and phenotype analyses. Additionally, we performed multivariable statistical analysis with adjustment for potential confounders and used visual summaries, including scatter plots, box plots, bar graphs, and matrix plots, to show data distribution and risk patterns.

However, several limitations should be acknowledged. First, the retrospective design precludes establishing causal relationships, and residual confounding remains possible despite our adjustments. Second, several thyroid dysfunction subclasses, particularly overt hypothyroidism and hyperthyroidism, included small numbers of patients; therefore, the combined association of thyroid dysfunction and ARR with target organ damage should be interpreted cautiously. Third, we did not have longitudinal data on all patients to assess how changes in thyroid function might affect ARR and target organ damage over time. Fourth, high‐sensitivity cardiac troponin was not consistently available and could not be incorporated into the primary cardiac damage assessment, although cardiac damage was defined using clinically meaningful structural and coronary criteria. Fifth, our population was drawn from a single medical center, which may limit generalizability, although the ethnic diversity partially mitigates this concern. Sixth, medication effects could not be completely eliminated, despite our adjustments and subgroup analyses.

Future research should include prospective studies examining how normalization of thyroid function affects ARR and target organ damage in PA patients, investigations into the molecular mechanisms underlying the observed correlations, and evaluations of whether thyroid function assessment improves risk stratification and treatment selection in PA.

## 5. Conclusion

In conclusion, our study demonstrates significant correlations between thyroid function parameters and the ARR in patients referred to for evaluation of suspected or confirmed PA, as well as associations of thyroid dysfunction and elevated ARR with target organ damage. These findings suggest that comprehensive thyroid function assessment may complement ARR‐based evaluation and improve risk stratification in PA‐related hypertension. Given the high prevalence of both endocrine abnormalities and their potential cardiovascular, cerebrovascular, and renal implications, integrated assessment of these systems represents a promising approach to optimizing management of these complex patients.

Thyroid function parameters, particularly higher TSH and lower FT4, were significantly associated with ARR in this cohort of patients referred to for evaluation of suspected or confirmed PA. Elevated ARR was consistently associated with cardiovascular, cerebrovascular, and renal target organ damage, while thyroid dysfunction was mainly associated with cardiovascular and cerebrovascular damage. These findings support integrated assessment of thyroid function and RAAS activity in patients with PA‐related hypertension, while prospective multicenter studies with complete high‐sensitivity troponin measurement and larger thyroid dysfunction subgroups are needed to validate these associations and refine risk prediction.

## Author Contributions

We declare that all listed authors have actively participated in the study and meet the requirements for authorship. Changjiang Deng designed the study, oversaw the definition of intellectual content, and is the guarantor of the study’s integrity. Yixin Xu managed the literature searches and analyses, Ying Pan prepared the paper, Tingting Wu edited the paper, Zhihui Jiang and Mingming Lv undertook the data acquisition and analysis, Yingying Zheng contributed to the statistical analysis, and Xiang Xie contributed to the correspondence and paper revision. All authors reviewed the manuscript.

## Funding

This study was funded by the Xinjiang Science and Technology, Tian Shan Yingcai Lingjun Project (2022TSYCLJ0029) and the Special Funds Program for Central Guiding Local Science and Technology Development (ZYYD2024CG07).

## Ethics Statement

The study was approved by the Institutional Review Board of The First Affiliated Hospital of Xinjiang Medical University (Approval Number: 240528‐09), and individual informed consent was waived due to the retrospective nature of the analysis. All procedures were performed in accordance with the ethical standards of the institutional research committee and the 1964 Declaration of Helsinki and its later amendments.

## Consent

Please see the Ethics Statement.

## Conflicts of Interest

The authors declare no conflicts of interest.

## Data Availability

The data that support the findings of this study are available from the corresponding author upon reasonable request.
